# Supplementary material for: Causal Relationship between Aging and Anorexia Nervosa: A White-Matter-Microstructure-Mediated Mendelian Randomization Analysis
Source: Biomedicines. 2024 Aug 16;12(8):1874. doi: 10.3390/biomedicines12081874 (PMC11351342; doi:10.3390/biomedicines12081874)
Supplement: Supplementary file 1 [file biomedicines-12-01874-s001.zip › Supplementary Figure/Figure S4/Singe SNP plot of MR analysis for TL to ubm-b-1560.pdf]

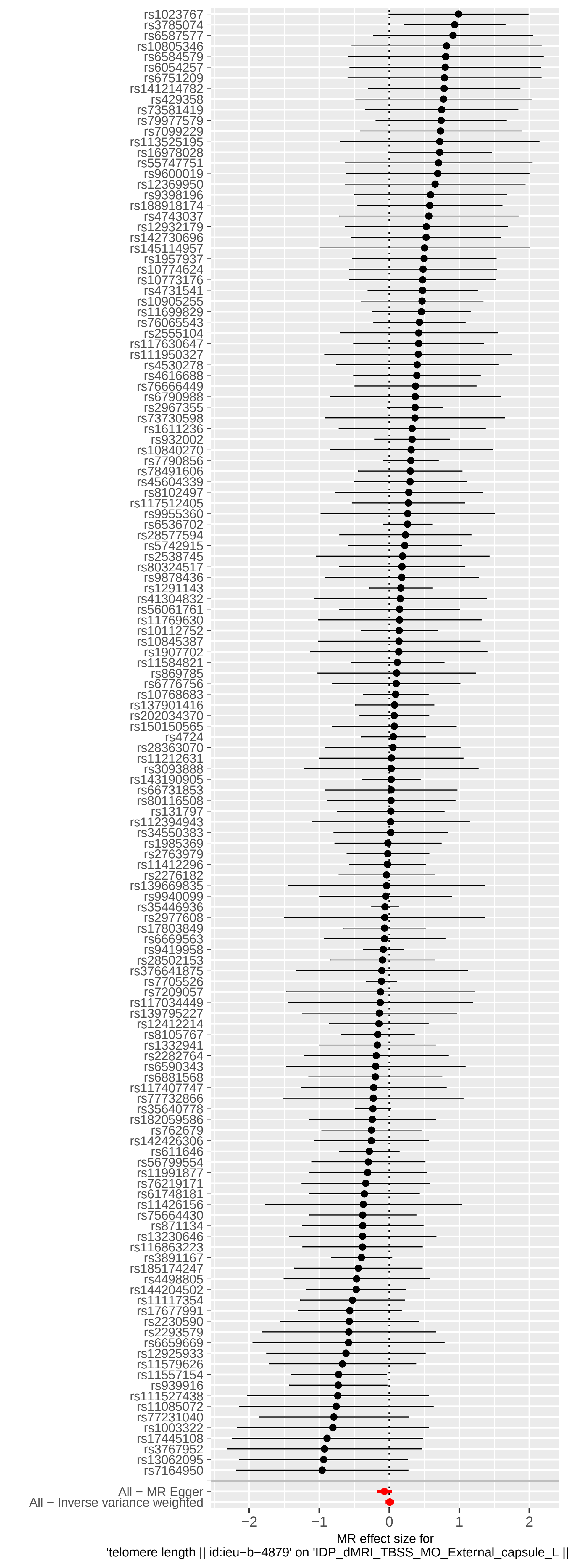
rs1023767
rs3785074
rs6587577
rs10805346
rs6584579
rs6054257
rs6751209
rs141214782
rs429358
rs73581419
rs79977579
rs7099229
rs113525195
rs16978028
rs55747751
rs9600019
rs12369950
rs9398196
rs188918174
rs4743037
rs12932179
rs142730696
rs145114957
rs1957937
rs10774624
rs10773176
rs4731541
rs10905255
rs11699829
rs76065543
rs2555104
rs117630647
rs111950327
rs4530278
rs4616688
rs76666449
rs6790988
rs2967355
rs73730598
rs1611236
rs932002
rs10840270
rs7790856
rs78491606
rs45604339
rs8102497
rs117512405
rs9955360
rs6536702
rs28577594
rs5742915
rs2538745
rs80324517
rs9878436
rs1291143
rs41304832
rs56061761
rs11769630
rs10112752
rs10845387
rs1907702
rs11584821
rs869785
rs6776756
rs10768683
rs137901416
rs202034370
rs150150565
rs4724
rs28363070
rs11212631
rs3093888
rs143190905
rs66731853
rs80116508
rs131797
rs112394943
rs34550383
rs1985369
rs2763979
rs11412296
rs2276182
rs139669835
rs9940099
rs35446936
rs2977608
rs17803849
rs6669563
rs9419958
rs28502153
rs376641875
rs7705526
rs7209057
rs117034449
rs139795227
rs12412214
rs8105767
rs1332941
rs2282764
rs6590343
rs6881568
rs117407747
rs77732866
rs35640778
rs182059586
rs762679
rs142426306
rs611646
rs56799554
rs11991877
rs76219171
rs61748181
rs11426156
rs75664430
rs871134
rs13230646
rs116863223
rs3891167
rs185174247
rs4498805
rs144204502
rs11117354
rs17677991
rs2230590
rs2293579
rs6659669
rs12925933
rs11579626
rs11557154
rs939916
rs111527438
rs11085072
rs77231040
rs1003322
rs17445108
rs3767952
rs13062095
rs7164950
All − MR Egger
All − Inverse variance weighted
−2
−1
0
1
2
MR effect size for
'telomere length || id:ieu−b−4879' on 'IDP_dMRI_TBSS_MO_External_capsule_L ||
